# Breaking Through: My Life in Science

**DOI:** 10.3201/eid3004.231656

**Published:** 2024-04

**Authors:** Sara C. Keller

**Affiliations:** Johns Hopkins University School of Medicine, Baltimore, Maryland, USA

**Keywords:** vaccines, mitochondrial RNA, biotechnology, books and media

In Breaking Through: My Life in Science, Dr. Katalin Karikó describes her fascinating, sometimes frustrating, and always inspiring journey from life in post–World War II communist Hungary to finally being recognized for her contributions toward successfully developing mitochondrial RNA (mRNA) technology that was used, among other things, to develop the Pfizer-BioNTech SARS-CoV-2 vaccine (Figure). Karikó’s autobiography was published just a week after she and her long-term collaborator, Dr. Drew Weissman, were awarded the 2023 Nobel Prize in physiology and medicine. The story takes us chronologically from growing up in rural 1950–1960s Hungary, through working as a researcher in academic medical centers in Philadelphia, Pennsylvania, USA, raising a two-time Olympic gold medalist rower, and finally to her current roles as a biochemist and researcher and senior vice-president at BioNTech company in Mainz, Germany. 

**Figure:**
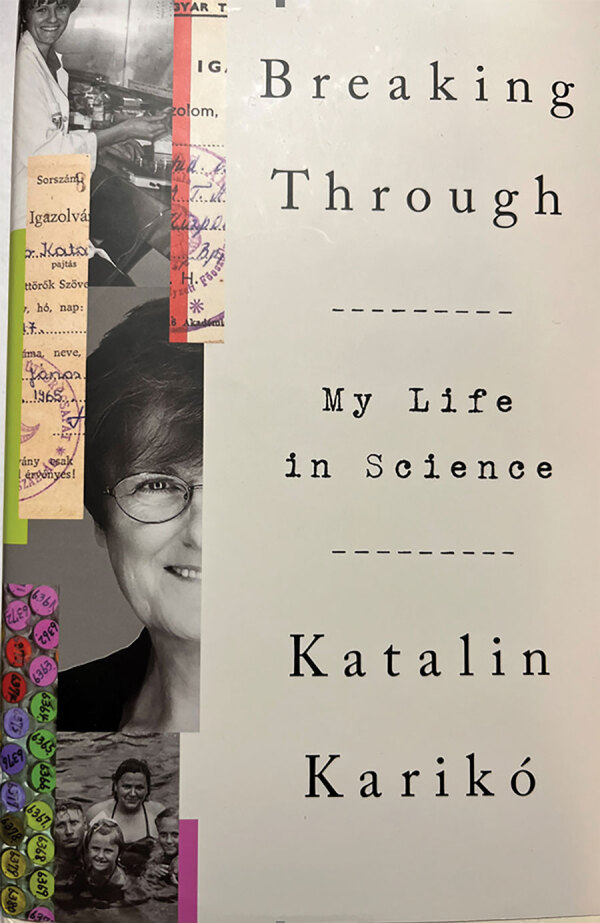
Breaking Through: My Life in Science

Although Karikó cautions readers that scientists can be notoriously bad at explaining things to nonscientists, she herself writes in an engagingly clear and simple style. Readers will be inspired by her description of growing up in a rural Hungarian town in an adobe hut with no indoor plumbing, limited electricity, and very little heat. Her mother was a bookkeeper and her father a butcher who sometimes got in trouble with local Communist Party leadership. What I found most moving was the persistence Karikó displayed in studying so hard in primary school that she placed nationally in biology competitions. Karikó notes that she received one of several spots in an extremely competitive university biology program in Hungary held specifically for students from less advantaged backgrounds. Karikó’s university educational experience and later scientific contributions illustrate the potential value of programs to assist academically qualified but economically challenged students (*1*,*2*). 

Upon arriving in the United States as a non–tenure track laboratory researcher at Temple University, Karikó entered an environment in which she faced seemingly insurmountable resistance. The male principal investigator at times screamed and threw objects at her. When she attempted to leave, he threatened to have her deported and blackballed. Karikó describes her decades of meticulous work researching mRNA during which time she applied for grants and submitted articles reporting her work to prestigious scientific journals, only to have those manuscripts rejected. Department heads reminded her continuously about her need to bring in funds. Karikó remained in nontenured positions until one day in 2013 she arrived at her laboratory at the University of Pennsylvania to find her equipment being moved out. She moved to the BioNTech company to continue her research and just 10 years later, she had won the Nobel Prize for scientific work that led directly to saving millions of lives. One wonders how much grants and promotion systems that reward creativity, meticulousness, and perseverance, not just rapid success and publication, could lead to more breakthroughs. 

Dr. Kariko’s story recounts her persistence over many obstacles in pursuit of goals springing from her love for science. Scientists, clinicians, and lay readers will all find this story a compelling read. 
